# Osteoclast-derived exosomal miR-378a-3p targets FTO-mediated m^6^A modification to promote osteogenesis and inhibit osteocyte apoptosis in alcohol-induced osteonecrosis of the femoral head

**DOI:** 10.3389/fcell.2026.1975355

**Published:** 2026-10-26

**Authors:** Canbin Zhao, Jinfu Liu, Dongfeng Chen, Donghui Guan, Liang Guo, Weiwei Wang, Qingyuan Xu, Hongcheng Tao, Chicheng Niu, Bin Sun, Jiang Shao, Ping Zeng

**Affiliations:** 1 The First College of Clinical Medicine, Guangxi University of Chinese Medicine, Nanning, Guangxi, China; 2 Department of Orthopedics,The First Affiliated Hospital of Guangxi University of Chinese Medicine, Nanning, Guangxi, China; 3 College of Traditional Chinese Medicine, Jinan University, Guangzhou, Guangdong, China; 4 The First Clinical Medical College, Shandong University of Traditional Chinese Medicine, Jinan, Shandong, China; 5 Department of Orthopedics, Affiliated Hospital of Shandong University of Traditional Chinese Medicine, Jinan, Shandong, China; 6 Sanya Hospital of Traditional Chinese Medicine, Sanya, Hainan, China

**Keywords:** alcohol-induced osteonecrosis of the femoral head, exosomes, FTO, miR-378a-3p, osteocyte apoptosis, osteogenesis

## Abstract

**Background:**

Alcohol-induced osteonecrosis of the femoral head (AIONFH) is associated with impaired bone formation and increased bone cell apoptosis. This study investigated the role of osteoclast-derived exosomal miR-378a-3p and the FTO-mediated m^6^A pathway in AIONFH.

**Methods:**

Rat bone marrow-derived macrophages (BMMs) were induced to form osteoclasts with or without ethanol. Exosomes were isolated, and differentially expressed miRNAs were identified by sequencing and RT-qPCR. The interaction between miR-378a-3p and FTO was examined using a dual-luciferase reporter assay. The effects of modified osteoclast-derived exosomes on BMSC osteogenic differentiation, apoptosis, FTO expression, and m^6^A levels were evaluated under ethanol exposure. An AIONFH rat model was used to assess the therapeutic effects of these exosomes *in vivo*.

**Results:**

Ethanol promoted osteoclast formation and reduced miR-378a-3p levels in osteoclast-derived exosomes. miR-378a-3p directly targeted the FTO 3′-UTR and reduced FTO expression. Under ethanol exposure, FTO expression increased, m^6^A levels decreased, osteogenic differentiation was inhibited, and apoptosis was increased. miR-378a-3p-enriched exosomes reversed these changes by reducing FTO expression, increasing m^6^A levels, promoting osteogenesis, and reducing apoptosis. In AIONFH rats, these exosomes improved trabecular bone structure, increased osteogenic marker expression, and reduced femoral head apoptosis. Exosomes with miR-378a-3p inhibition produced opposite effects.

**Conclusion:**

Osteoclast-derived exosomal miR-378a-3p protects against alcohol-induced bone damage by targeting FTO and regulating m^6^A modification. The miR-378a-3p/FTO/m^6^A pathway may be a potential therapeutic target for AIONFH.

## 1 Introduction

Chronic and excessive alcohol consumption leads to alcohol-induced osteonecrosis of the femoral head (AIONFH), a serious bone and joint disorder that mainly affects heavy drinkers in young and middle-aged populations (Konarski et al., 2023; Yoon et al., 2019). Because early-stage AIONFH has few symptoms and develops slowly, it is often misdiagnosed or missed. As a result, most patients are only diagnosed when the disease has reached moderate or severe stages, with permanent structural damage including femoral head collapse and reduced joint movement, which seriously affects their quality of life (Parikh et al., 2026; Pang et al., 2025).

The main cause of AIONFH is disrupted bone metabolism (Liao et al., 2022; Hong et al., 2019). In healthy conditions, the structural integrity of the femoral head bone network is maintained by a balance between bone formation (by osteoblasts) and bone resorption (by osteoclasts) (Wu et al., 2020; Wang et al., 2023). Long-term alcohol exposure breaks this balance. It blocks osteoblast growth and differentiation, which reduces bone formation, while also abnormally activating osteoclasts, which increases bone resorption. In addition, alcohol causes severe osteocyte death, which further disrupts bone balance and leads to progressive bone death and disease progression (Maurel et al., 2012; Tan et al., 2021).

Exosomes are important carriers for cell-to-cell communication. They transport functional molecules, especially miRNAs, to regulate bone cell activities and maintain metabolic balance (Tang et al., 2024; Shuai et al., 2026). However, alcohol can significantly change the miRNA expression patterns in osteoclast-derived exosomes. Through paracrine signaling, these altered exosomes damage osteoblast function and worsen bone structural damage (Wang et al., 2025; Ye et al., 2024; Sun et al., 2016). In bone remodeling, N^6^-methyladenosine (m^6^A) RNA modification is an important epigenetic regulator. Fat mass and obesity-associated protein (FTO) is a key m^6^A demethylase that controls osteogenic differentiation and apoptosis by changing the m^6^A methylation levels of specific target genes (Xie et al., 2026). Previous studies show that exosomal miRNAs can directly target FTO, which affects m^6^A modification levels and changes bone cell functions (Huang et al., 2023). However, the exact regulatory mechanism of the exosomal miRNA-FTO-m^6^A pathway in AIONFH metabolic imbalance is still unclear.

To address this question, our study analyzed the miRNA expression profiles of osteoclast-derived exosomes grown in an alcohol environment to identify key functional miRNAs. Using both *in vitro* and *in vivo* models, we studied the molecular mechanisms by which these miRNAs restore bone metabolism balance by targeting FTO-mediated m^6^A modifications. This research aims to explain the important role of this regulatory pathway in AIONFH development and progression, providing a strong theoretical basis for understanding its pathology and improving clinical treatment strategies.

## 2 Materials and methods

### 2.1 Osteoclast induction and morphological observation of BMMs

Rat bone marrow-derived macrophages (BMMs; Procell Life Science and Technology Co., Ltd., Wuhan, China) were cultured *in vitro*. Cells were divided into the standard induction group (BMMs + RANKL) and alcohol-exposed group (BMMs + RANKL + ethanol). Cells in the standard group were cultured in osteoclast induction medium (Sunncell Biosciences Inc., Wuhan, China). For the alcohol-exposed group, the same induction medium supplemented with 50 mM ethanol (Shanghai Yuanye Bio-Technology Co., Ltd., Shanghai, China) was used to mimic the alcoholic microenvironment (Jia et al., 2019). Both groups were continuously cultured for 7 days. Following induction, morphological changes were observed. The effects of ethanol on the maturation and fusion of BMMs into osteoclasts were evaluated using a TRAP staining kit and phalloidin staining kit (both from Servicebio, Wuhan, China).

### 2.2 Extraction and identification of osteoclast-derived exosomes

After osteoclast induction, culture supernatants were collected and exosomes were isolated by differential ultracentrifugation. Supernatants were sequentially centrifuged at 2,000 × g (30 min, 4 °C) to remove cells and debris, at 10,000 × g (45 min, 4 °C) to deplete microvesicles, filtered through a 0.45 μm filter (Millipore), and ultracentrifuged at 100,000 × g (70 min, 4 °C). Pellets were washed once with ice-cold PBS (Servicebio, Wuhan, China) by ultracentrifugation (100,000 × g, 70 min, 4 °C), resuspended in 100 μL PBS, and quantified by BCA assay (Thermo Fisher Scientific). Exosomes were stored at −80 °C. Characterization included transmission electron microscopy (Hitachi, Tokyo, Japan), nanoparticle tracking analysis (NTA), and Western blot for CD63, TSG101, and Calnexin.

### 2.3 Sequencing and validation of osteoclast-derived exosomal miRNAs

High-quality exosomal miRNAs were isolated from purified osteoclast-derived exosome samples of each group following a standard workflow including cell lysis, organic-phase separation, spin-column purification, and final elution. RNA concentration and purity were determined using a fluorometric quantitation detector (Roche, Basel, Switzerland). Small-RNA library construction and sequencing were performed by Xiamen Life Origin Biotech Co., Ltd (Xiamen, China) to screen key differentially expressed functional miRNAs linked to alcohol-provoked bone metabolic disturbance. Candidate miRNAs obtained from sequencing analysis were further validated by real-time quantitative polymerase chain reaction (RT-qPCR).

### 2.4 Dual-luciferase reporter assay

The TargetScan database (McGeary et al., 2019) (https://www.targetscan.org/vert_80/) was utilized to predict potential binding sites between FTO and the identified functional miRNAs. We constructed pmirGLO dual-luciferase reporter plasmids (Thermo Fisher Scientific, Wuxi, China) harboring either the wild-type or mutant 3′-UTR sequences of FTO. Following this, HEK293T cells were co-transfected with the respective functional miRNA mimics and the recombinant plasmids (wild-type or mutant). Post-transfection and incubation, the relative luciferase activities were determined by measuring and comparing the firefly and Renilla luciferase signals across the groups. Details concerning the pmirGLO dual-luciferase vectors are provided in Supplementary Table S1.

### 2.5 Overexpression and knockdown of functional miRNAs in BMMs

Plasmid vectors designed for overexpression (miR-mimic) and knockdown (miR-inhibitor) (Thermo Fisher Scientific, Wuxi, China) were engineered to target the specific miRNA that exhibited prominent expression alterations and contained an FTO binding site. BMMs were transfected with these vectors along with their respective negative controls (miR-mimic-NC and miR-inhibitor-NC) (Supplementary Figure S1). Subsequently, these transfected BMMs were driven toward osteoclast differentiation under alcoholic conditions, after which their osteoclast exosomes were harvested for downstream applications.

### 2.6 Overexpression and knockdown of FTO in BMSCs

For the experiments involving bone marrow mesenchymal stem cells (BMSCs; Procell Life Science and Technology Co., Ltd., Wuhan, China), we generated an FTO overexpression recombinant plasmid (oe-FTO), small interfering RNA for FTO knockdown (si-FTO), and corresponding negative control sequences (Thermo Fisher Scientific, Wuxi, China). BMSCs were then transfected with oe-FTO, si-FTO, or their negative controls (Vector-NC and si-NC) (Supplementary Figure S2). To initiate osteogenic differentiation, the transfected BMSCs were cultured in an osteogenic induction medium (Procell Life Science and Technology Co., Ltd., Wuhan, China) supplemented with 50 mM ethanol to simulate an alcoholic milieu (Yu H. et al., 2020). Finally, these cells were treated with the modified osteoclast exosomes obtained in Section 2.5.

### 2.7 Exosome endocytosis tracking assay

Exosome membranes were labeled with DiO and encapsulated miRNAs with FAM. After removing free dye, dual-labeled exosomes were co-incubated with BMSCs in confocal glass-bottom dishes at 37 °C. Cells were then fixed, and counterstained with DAPI. Intracellular localization of DiO and FAM signals was observed by laser scanning confocal microscopy.

### 2.8 Determination of BMSC apoptosis

Apoptotic cells were identified using a TUNEL staining kit (Servicebio, Wuhan, China). Serum-free α-MEM containing the specific reagents was used to culture the cells seeded onto cover glasses in 24-well plates for 48 h. Subsequently, TUNEL staining was performed according to the manufacturer’s instructions, followed by sealing and observation under a fluorescence microscope. Green staining indicated TUNEL-positive cells, and three randomly selected fields were analyzed to calculate the percentage of positive cells.

### 2.9 Alkaline phosphatase (ALP) staining

BMSCs were cultured in 24-well plates at 37 °C in a humidified incubator containing 5% CO_2_. The cells were maintained in basic medium for 2 days, after which the medium was replaced with osteogenic induction medium. Following 7 days of osteogenic induction, ALP staining was conducted with a BCIP/NBT ALP colorimetric kit (Servicebio, Wuhan, China). After staining, micrographs were obtained for further observation.

### 2.10 Alizarin red S (ARS) staining

The BMSCs were seeded and maintained under the same conditions described in the ALP staining procedure. After 21 days of osteogenic induction, ARS staining was carried out to evaluate the formation of mineralized nodules. Briefly, after fixing, the samples were then incubated with ARS solution (pH 4.2, Servicebio, Wuhan, China) at 37 °C for 30 min. After staining, distilled water was used to wash away the unbound dye, and representative micrographs were acquired.

### 2.11 RT-qPCR assay

Total RNA was extracted from BMSCs and exosomal small RNA from osteoclast-derived exosomes using TRIzol reagent (Servicebio, Wuhan, China), and reverse-transcribed into cDNA. RT-qPCR was performed with SYBR Green (GeneCopoeia, United States of America) on a LightCycler 480 system (Roche, Switzerland). Osteogenic markers (ALP, OCN, Collagen-1), apoptosis-related genes (Bax, Bcl-2, Caspase-3), FTO, and exosomal miRNAs were quantified. GAPDH and U6 snRNA served as internal controls for mRNA and miRNA, respectively. Relative expression was calculated by the 2^−ΔΔCT^ method. Primer sequences are listed in Supplementary Tables S2–S4.

### 2.12 Quantification of m^6^A methylation

Total RNA was isolated using TRIzol (Servicebio, Wuhan, China). The quality of RNA was analyzed using a NanoDrop™ Spectrophotometer (Thermo Fisher Scientific Inc.). Subsequently, the EpiQuik m^6^A RNA Methylation Quantification Kit (Brooklyn, NY, United States of America) was used to determine the m^6^A content in total RNA (Wang X. K. et al., 2021). Briefly, 200 ng of RNA was first added to each well for coating, followed by the sequential addition of capture and detection antibodies at the appropriate concentrations according to the manufacturer’s instructions. A standard curve was generated from the absorbance values measured at 450 nm for each well to quantify the m^6^A levels. All samples were assessed in triplicate.

### 2.13 Western blot assay

RIPA buffer (Servicebio, Wuhan, China) supplemented with protease and phosphatase inhibitors was used to extract total protein, which was subsequently quantified using a BCA assay (Servicebio). A total of 30–60 μg of protein per lane was separated by 10% SDS-PAGE and transferred onto PVDF membranes (Servicebio). Primary antibodies against TSG101, CD63, Calnexin, ALP, OCN, collagen I, and β-actin (1:1,000; HUABIO, Hangzhou, China) were incubated with the blocked membranes overnight at 4 °C, followed by HRP-conjugated secondary antibodies (1:5,000; Beyotime, Nanjing, China). Signals were detected using BeyoECL Star (Beyotime) on a ChemiDoc Touch system (Bio-Rad, Hercules, CA, United States of America), and band intensities were quantified by ImageJ (NIH, Bethesda, MD, United States of America).

### 2.14 Animal modeling, grouping, and intervention

A total of 48 specific pathogen-free (SPF) Sprague-Dawley (SD) rats (6 weeks old, 220 ± 20 g, equal gender distribution) were procured from Hunan Slike Jingda Experimental Animal Co., Ltd (Hunan, China). The corresponding licenses included Certificate No.: 430727251103174853 and License No.: SCXK (Xiang) 2024-0009. These animals were maintained at the Animal Experimental Center affiliated with Guangxi University of Chinese Medicine (Experimental Animal Use License No.: SYXK (Gui) 2024-0004). To construct the AIONFH *in vivo* model, the subjects were continuously administered an alcohol-infused liquid diet (Bio-Serv LD82) over a 12-week period (Cheng et al., 2025). Following successful modeling, the rats were randomly allocated into four groups (n = 12 per group): (1) AIONFH model group, receiving tail vein injections of PBS; (2) AIONFH + Exo-NC group, receiving standard osteoclast exosomes; (3) AIONFH + Exo-miR-mimic group, receiving 100 μg of osteoclast exosomes overexpressing miR-378a-3p; and (4) AIONFH + Exo-miR-inhibitor group, receiving 100 μg of osteoclast exosomes with miR-378a-3p knockdown. For exosome interventions, a weekly dosage of 100 μg suspended in 200 μL of PBS was administered via tail vein injection over 6 weeks (Le et al., 2025).

### 2.15 Micro-CT analysis

Femoral specimens were fixed in 4% paraformaldehyde and scanned by Micro-CT. Three-dimensional reconstructions were generated using multimodal 3D visualization software (Pingsheng Medical Technology, Suzhou, China), and bone parameters were analyzed with CT Analyzer software (Bruker, Kontich, Belgium). The subchondral trabecular region of the femoral head was defined as the region of interest (ROI), and bone surface (BS), bone volume (BV), total tissue volume (TV), and bone volume fraction (BV/TV) were quantified.

### 2.16 Histological staining

To visualize pathological changes and evaluate bone-matrix restoration under different exosome treatments, the prepared paraffin-embedded femoral head sections were stained with several commercial kits (all from Servicebio, Wuhan, China), including Hematoxylin-Eosin (HE) and Masson’s trichrome staining.

### 2.17 Immunofluorescence assay

After dewaxing, sections underwent antigen retrieval by microwaving for 20 min, followed by PBS washing and blocking. Sections were incubated overnight at 4 °C with primary antibodies against FTO, ALP, and OCN, then with fluorescent secondary antibodies, and counterstained with DAPI. Images were captured under a fluorescence microscope (Olympus, Japan).

### 2.18 TUNEL staining

Osteocyte apoptosis was evaluated on decalcified paraffin-embedded femoral head sections using a commercial TUNEL detection kit (Servicebio, Wuhan, China) following the manufacturer’s protocol. The subchondral trabecular region of the femoral head was set as the region of interest (ROI). Fluorescence images were acquired with an inverted fluorescence microscope. TUNEL-positive osteocytes were counted in three random high-power fields per section within the ROI to determine the anti-apoptotic efficacy of different exosomal treatments.

### 2.19 Immunohistochemical staining

Deparaffinization, rehydration, and antigen retrieval were performed on the tissue sections. Following blocking, primary antibodies against Caspase-3, Bcl-2, Bax, and FTO were applied to the sections and incubated, after which the appropriate secondary antibodies were applied. Sections were counterstained with hematoxylin and mounted. An optical microscope (Olympus, Tokyo, Japan) was used for image acquisition, and ImageJ software (NIH, Bethesda, MD, United States of America) was used for image analysis.

### 2.20 Statistical analysis

GraphPad Prism 9 software was utilized to execute all statistical evaluations and generate visualizations. The quantitative results are presented as the mean ± standard deviation (SD). To assess data normality, the Shapiro-Wilk test was applied, whereas Levene’s test evaluated variance homogeneity across the cohorts. If the multi-group datasets met the criteria for normal distribution and homogeneous variance, a one-way analysis of variance (ANOVA) was deployed, paired with Tukey’s *post hoc* test for group-to-group comparisons. In instances where normal distribution was present but variance was heterogeneous, Welch’s ANOVA accompanied by Dunnett’s T3 *post hoc* test was applied. Non-normally distributed datasets were scrutinized utilizing the Kruskal–Wallis rank-sum test, incorporating a Bonferroni correction for subsequent pairwise analyses. Statistical significance was designated at a two-tailed P-value threshold of <0.05.

## 3 Results

### 3.1 Characteristics of osteoclast-derived exosomal miR-378a-3p

We treated BMMs-derived osteoclast differentiation cultures with ethanol to evaluate its effects on osteoclast formation. From the results of tartrate-resistant acid phosphatase (TRAP) and Phalloidin staining, a marked increase in the number of osteoclasts was observed, accompanied by enlarged TRAP-positive cell bodies, increased F-actin bands, and a greater number of nuclei in the ethanol group (Figure 1A). Transmission electron microscopy revealed representative exosomes, while particle-size analysis showed that most particles had a diameter of approximately 70 nm (Figure 1B). Regardless of the presence or absence of ethanol treatment, the membrane characteristic proteins TSG101 and CD63 were positive, whereas Calnexin was negative in BMM-derived osteoclast exosomes (Figure 1C). Total RNA extracted from the abovementioned two groups was subjected to high-throughput sequencing. We observed that miR-378a-3p was markedly downregulated in exosomes following ethanol stimulation (Figure 1D). Consistently, RT-qPCR validation confirmed that the exosomal level of miR-378a-3p was significantly decreased by ethanol treatment (Figure 1E).

**FIGURE 1:**
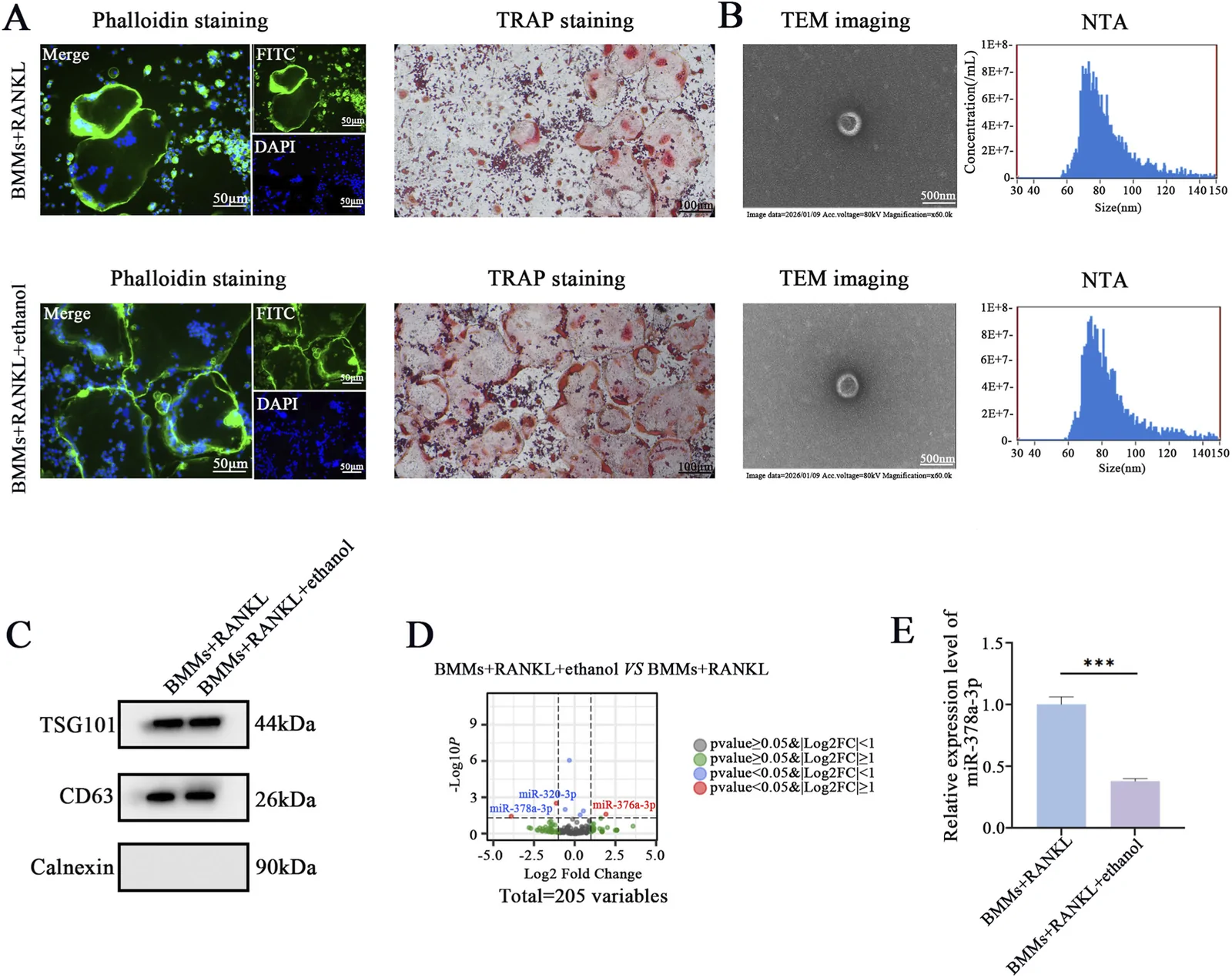
Characteristics of osteoclast-derived exosomal miR-378a-3p. **(A)** Phalloidin (FITC, green) and DAPI (blue) staining of F-actin cytoskeleton and TRAP staining (red) of RANKL-induced osteoclasts from BMMs treated with or without ethanol. Scale bars: 50 μm (Phalloidin), 100 μm (TRAP). **(B)** TEM morphology (left, scale bar = 500 nm) and NTA size distribution (right) of exosomes isolated from culture supernatants. **(C)** Western blot showing positive exosome markers TSG101 and CD63, with negative Calnexin. **(D)** Volcano plot of differentially expressed exosomal miRNAs (BMMs + RANKL + ethanol vs. BMMs + RANKL; 205 miRNAs total). Red dots: p < 0.05 and |Log2FC| ≥ 1. Key miRNAs labeled. **(E)** RT-qPCR validation of exosomal miR-378a-3p. n = 3; data are mean ± SD; ***P < 0.001.

### 3.2 Osteoclast-derived exosomal miR-378a-3p represses FTO to promote the osteogenic differentiation of BMSCs

The TargetScan database was used to screen for potential target genes of miR-378a-3p. There were potential binding sites on the 3′-UTR of FTO that were complementary to miR-378a-3p. Sanger sequencing was performed to verify the wild-type and mutant constructs of FTO (Figure 2A). The luciferase activity assay showed that miR-378a-3p mimic significantly decreased the activity of FTO 3′-UTR (Figure 2B). Next, the immunofluorescence assay demonstrated that exosomes and miR-378a-3p from osteoclasts could be absorbed by BMSCs (Figure 2C). In addition, BMSCs transfected with oe-FTO were treated with ethanol in the presence or absence of miR-378a-3p-overexpressing osteoclast-derived exosomes. Experimental results demonstrated that ethanol significantly inhibited osteogenic differentiation. Treatment with osteoclast-derived exosomes and FTO overexpression further aggravated this inhibitory effect. Conversely, osteoclast-derived exosomes overexpressing miR-378a-3p reversed the above inhibitory effects (Figures 2D,E). Western blotting revealed that expression levels of Collagen-1, FTO, ALP, and OCN further strengthened this evidence (Figures 2F,G).

**FIGURE 2:**
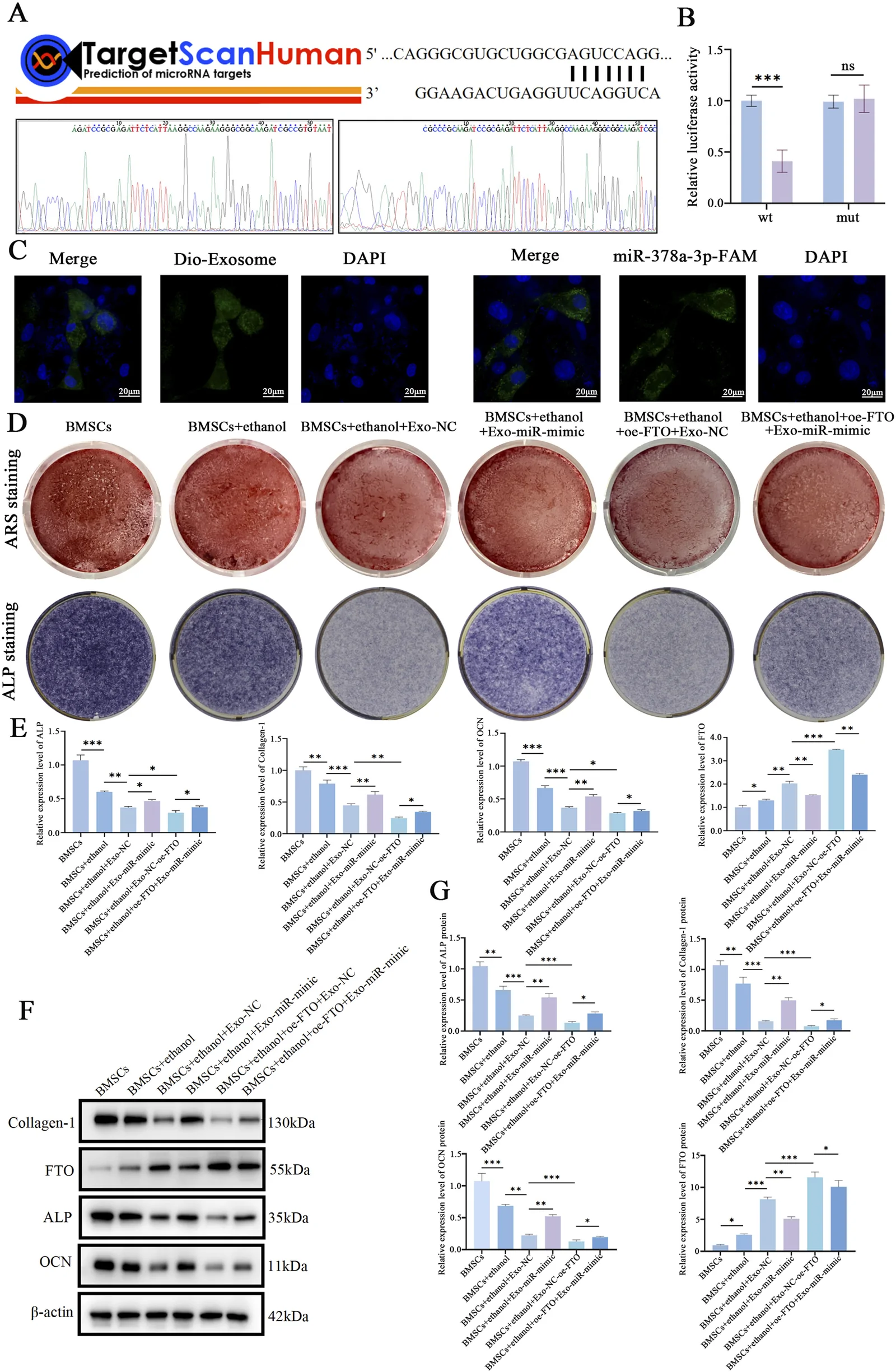
Osteoclast-derived exosomal miR-378a-3p represses FTO to promote the osteogenic differentiation of BMSCs. **(A)** TargetScanHuman prediction of the binding sites between miR-378a-3p and FTO 3′-UTR, with Sanger sequencing verification of wild-type and mutant constructs. **(B)** Dual-luciferase reporter assay in HEK293T cells co-transfected with miR-378a-3p mimic and wild-type (wt) or mutant (mut) FTO 3′-UTR reporters. **(C)** Internalization of DiO-labeled exosomes and FAM-labeled miR-378a-3p (green) into BMSCs observed by confocal microscopy; DAPI (blue) stains nuclei. Scale bar = 20 μm. **(D)** ARS (top) and ALP (bottom) staining. **(E)** RT-qPCR analysis of mRNA expression of ALP, Collagen-1, OCN, and FTO in BMSCs. **(F)** Western blot showing protein levels of Collagen-1, FTO, ALP, and OCN in BMSCs; β-actin serves as the loading control. **(G)** Quantification of the relative protein levels of ALP, Collagen-1, OCN, and FTO from **(F)** n = 3, data are mean ± SD; *P < 0.05, **P < 0.01, ***P < 0.001, ns, not significant.

### 3.3 FTO negatively regulates the osteogenic differentiation capacity of BMSCs

To determine the role of FTO in osteogenic differentiation, we knocked down and overexpressed FTO. RT-qPCR verified that si-FTO efficiently knocked down FTO expression, whereas oe-FTO induced robust FTO overexpression. Meanwhile, we examined the m^6^A modification function of FTO and found that the total m^6^A level in BMSCs was significantly increased after FTO knockdown (Figures 3A,C). Next, we detected the regulatory effect of FTO on osteogenic differentiation of BMSCs. The results showed that ethanol inhibited osteogenic differentiation, whereas this inhibitory effect was attenuated after FTO knockdown under ethanol conditions, as evidenced by enhanced ALP and ARS staining and increased mRNA and protein expression of osteogenic markers including ALP, OCN and Collagen-1. Conversely, FTO overexpression yielded the opposite results (Figures 3B–D). These results indicated that FTO negatively regulated osteogenic differentiation.

**FIGURE 3:**
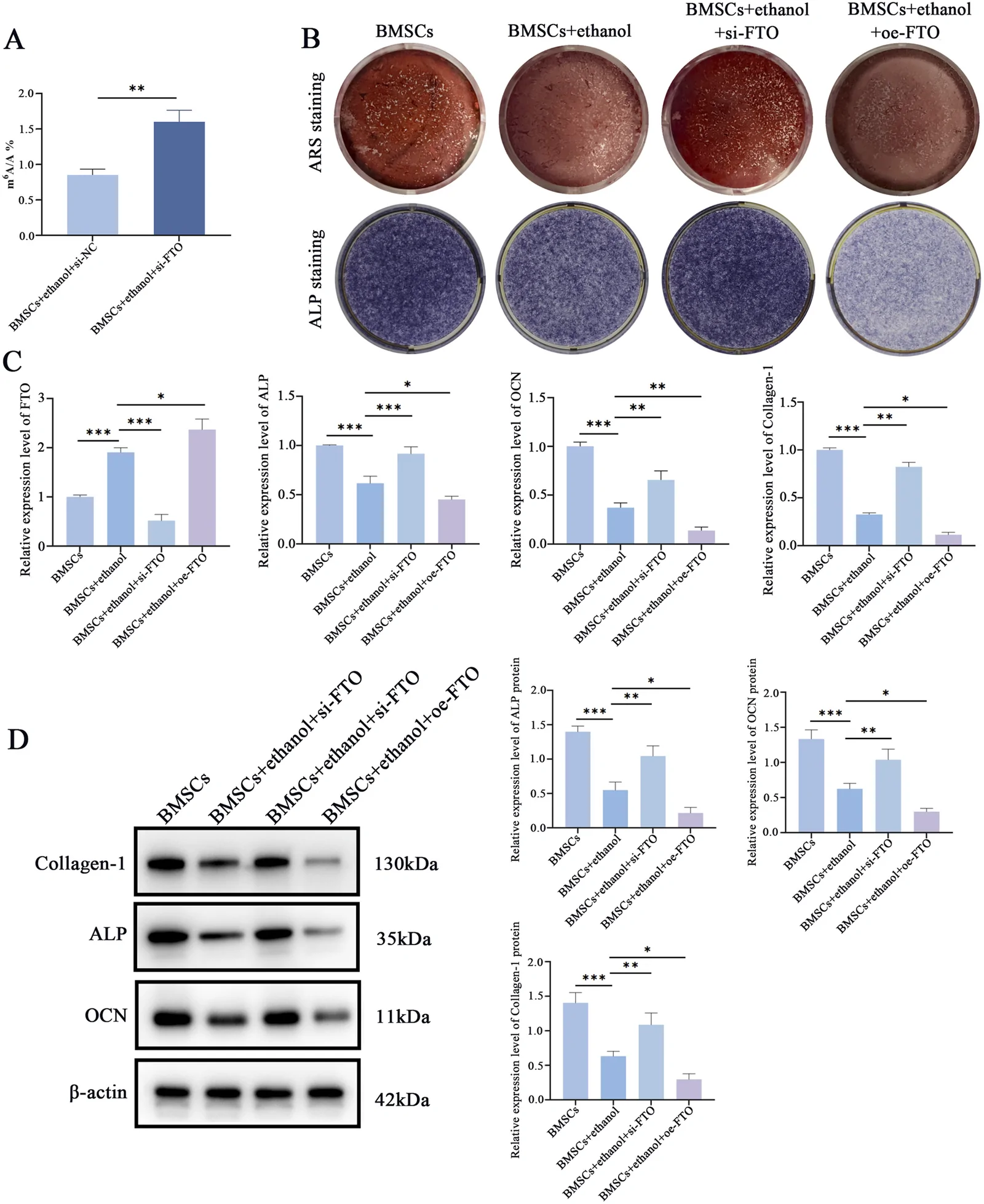
The m^6^A demethylase FTO negatively regulates the osteogenic differentiation of BMSCs *in vitro.* **(A)** m^6^A/A ratio in BMSCs transfected with si-NC or si-FTO under ethanol treatment. **(B)** ARS (top) and ALP (bottom) staining of BMSCs under four conditions: control, ethanol, ethanol + si-FTO, and ethanol + oe-FTO. **(C)** RT-qPCR analysis of mRNA expression of FTO, ALP, OCN, and Collagen-1 across the four groups. **(D)** Western blot showing protein levels of Collagen-1, ALP, and OCN across the four groups; β-actin serves as the loading control. Right: quantification of relative protein levels. n = 3, data are mean ± SD; *P < 0.05, **P < 0.01, ***P < 0.001.

### 3.4 Osteoclast-derived exosomal miR-378a-3p inhibits apoptosis in BMSCs via FTO

To further elucidate the role of miR-378a-3p in osteogenic differentiation, we co-cultured BMSCs transfected with oe-FTO with osteoclast-derived exosomes overexpressing miR-378a-3p, and evaluated apoptosis in BMSCs. TUNEL staining showed that Exo-NC intervention increased the number of TUNEL-positive cells in BMSCs under ethanol conditions, and this effect was further aggravated by oe-FTO treatment. In contrast, osteoclast-derived exosomal miR-378a-3p treatment reversed this effect, indicating that miR-378a-3p from osteoclast-derived exosomes effectively attenuated BMSC apoptosis (Figure 4A). During this process, the mRNA expression levels of FTO, Caspase-3 and the Bax/Bcl-2 ratio changed in parallel with the number of TUNEL-positive BMSCs (Figure 4B).

**FIGURE 4:**
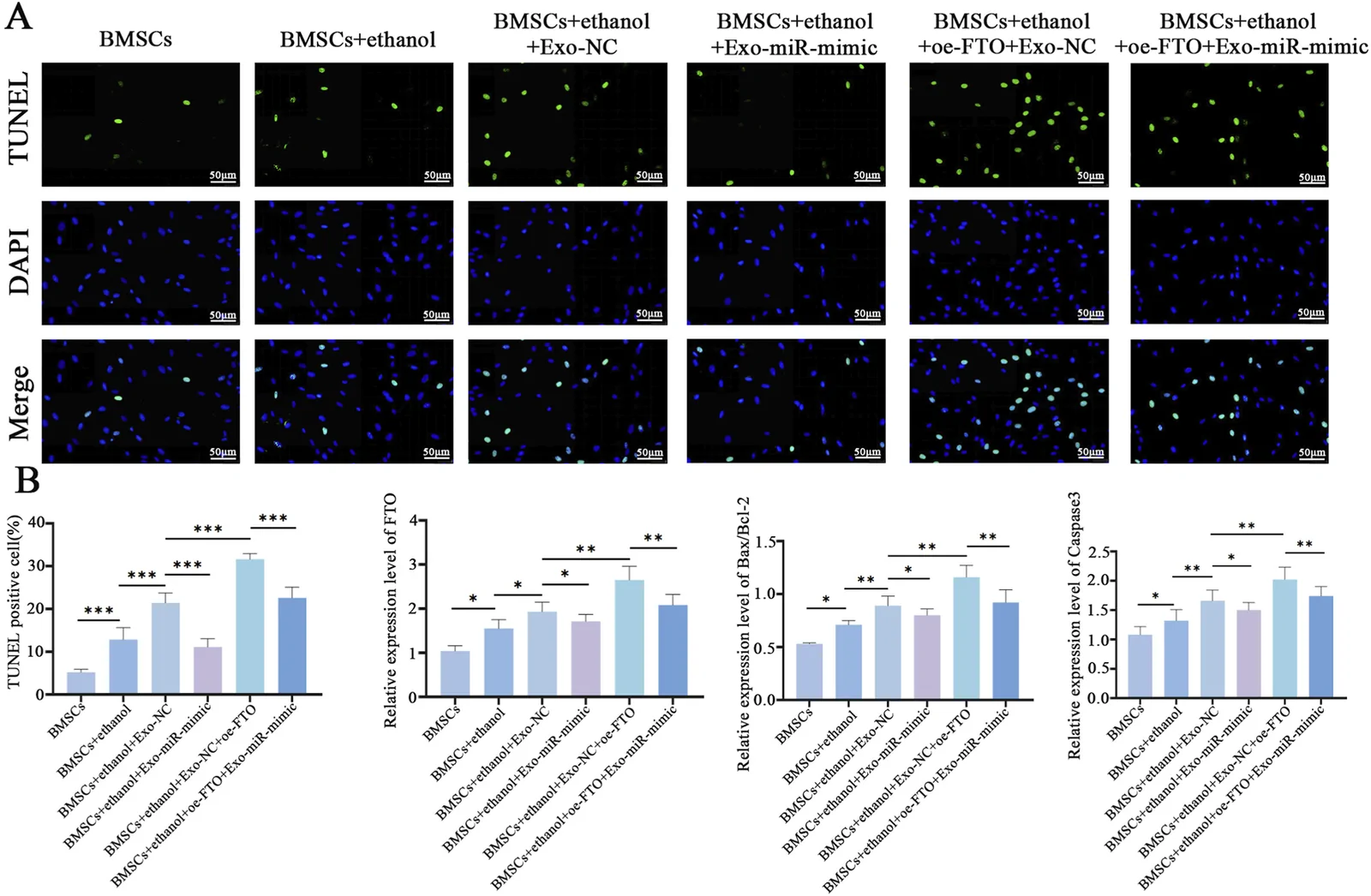
Osteoclast-derived exosomal miR-378a-3p inhibits apoptosis in BMSCs via FTO. **(A)** TUNEL staining (green) with DAPI nuclear counterstaining (blue) and merged images of BMSCs under six treatment conditions: control, ethanol, ethanol + Exo-NC, ethanol + Exo-miR-mimic, ethanol + oe-FTO + Exo-NC, and ethanol + oe-FTO + Exo-miR-mimic. Scale bar = 50 μm. **(B)** Quantification of TUNEL-positive cells (left), and relative mRNA expression levels of FTO, Bax/Bcl-2 ratio, and Caspase-3 across the six groups. n = 3, data are mean ± SD; *P < 0.05, **P < 0.01, ***P < 0.001.

### 3.5 miR-378a-3p in exosomes displays a clear rescue effect on AIONFH

To evaluate the therapeutic potential of osteoclast-derived exosomal miR-378a-3p in AIONFH, we conducted an *in vivo* experiment. Micro-CT imaging revealed that, compared with the AIONFH group, Exo-NC intervention resulted in abnormal femoral head morphology, significant trabecular bone loss and collapse, accompanied by marked decreases in bone parameters including BS, BV, TV and BV/TV. Compared with Exo-NC intervention, Exo-miR-inhibitor intervention exacerbated these changes, whereas Exo-miR-mimic intervention alleviated these changes (Figures 5A,B). Histological changes in AIONFH after different treatments were also assessed. H&E and Masson staining verified that Exo-miR-mimic attenuated AIONFH and enhanced calcification of new bone tissue in the weight-bearing region of the femoral head (Figure 5C). In addition, immunofluorescence staining was performed to examine the expression of osteogenic markers such as OCN and ALP. The results showed that, compared with the AIONFH group, the Exo-NC group exhibited a marked reduction in OCN-positive and ALP-positive cells in the rat femoral head, which was further aggravated by Exo-miR-inhibitor intervention. Compared with the Exo-NC group, the Exo-miR-mimic group showed an increased number of positive cells, while FTO exhibited the opposite trend (Figures 5D,E). Taken together, these findings suggest that osteoclast-derived exosomal miR-378a-3p promotes osteogenesis and prevents AIONFH by inhibiting FTO.

**FIGURE 5:**
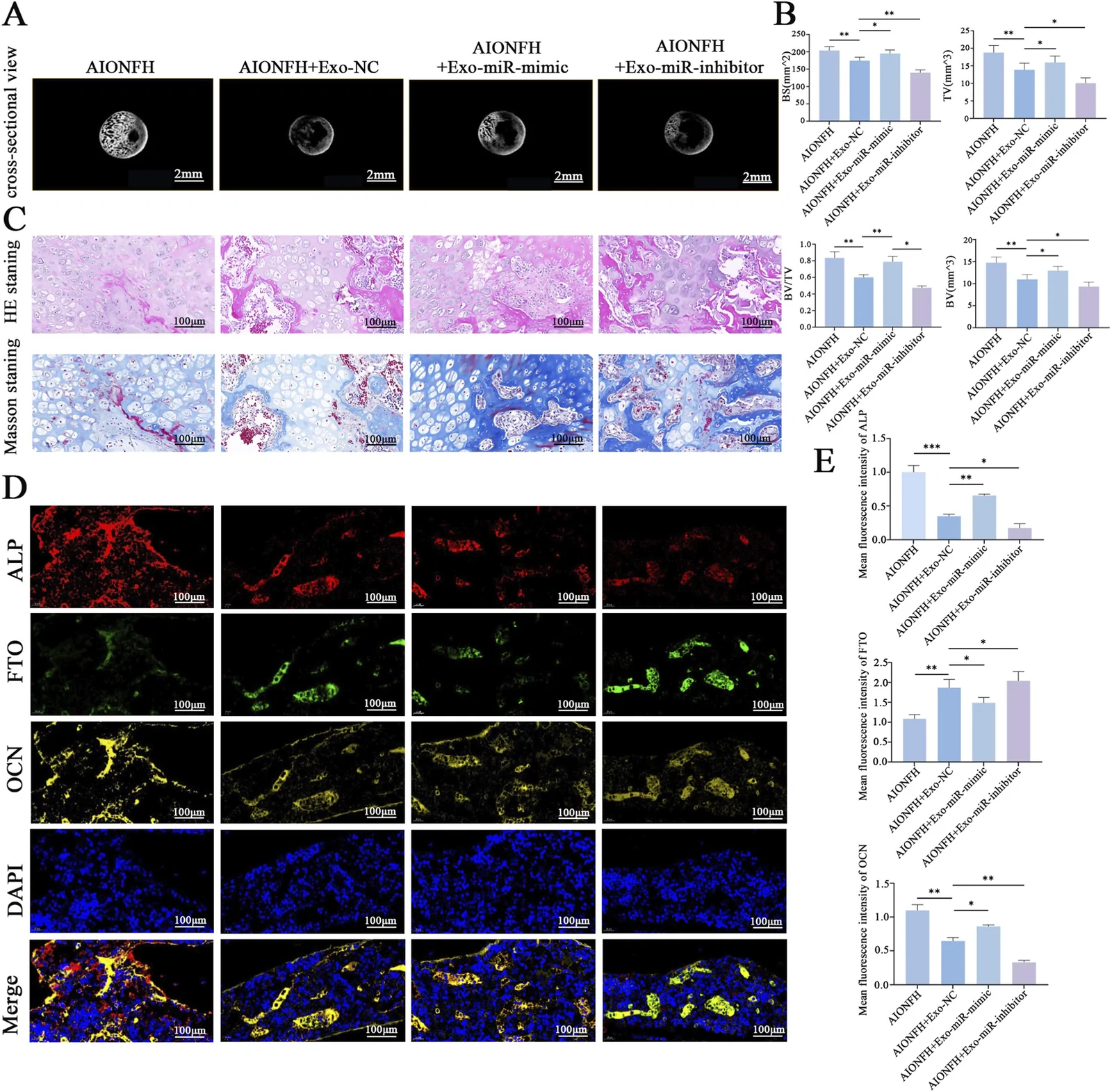
Osteoclast-derived exosomal miR-378a-3p ameliorates AIONFH by promoting osteogenesis *in vivo*. **(A)** Micro-CT cross-sectional images of femoral heads from four groups: AIONFH, AIONFH + Exo-NC, AIONFH + Exo-miR-mimic, and AIONFH + Exo-miR-inhibitor. Scale bar = 2 mm. **(B)** Quantitative analysis of BS, BV, TV, BV/TV from Micro-CT. **(C)** H&E staining (top) and Masson trichrome staining (bottom) of femoral head tissue across the four groups. Scale bar = 100 μm. **(D)** Immunofluorescence staining of ALP (red), FTO (green), OCN (yellow), and DAPI (blue), with merged images. Scale bar = 100 μm. **(E)** Mean fluorescence intensity quantification of ALP, FTO, and OCN. n = 3; data are mean ± SD; *P < 0.05, **P < 0.01, ***P < 0.001.

Furthermore, to validate the mechanisms mentioned in the *in vitro* experiments, apoptosis-related indicators were measured. TUNEL staining showed that, compared with the AIONFH group, the AIONFH + Exo-NC group exhibited a significant increase in apoptotic cells, which was further aggravated in the AIONFH + Exo-miR-inhibitor group. In contrast, compared with the AIONFH + Exo-NC group, the AIONFH + Exo-miR-mimic group showed a reduction in apoptotic cells (Figures 6A,C). Immunohistochemical staining revealed that the expression levels of FTO, the Bax/Bcl-2 ratio and Caspase-3 exhibited the same trend as the number of apoptotic cells (Figures 6B,D).

**FIGURE 6:**
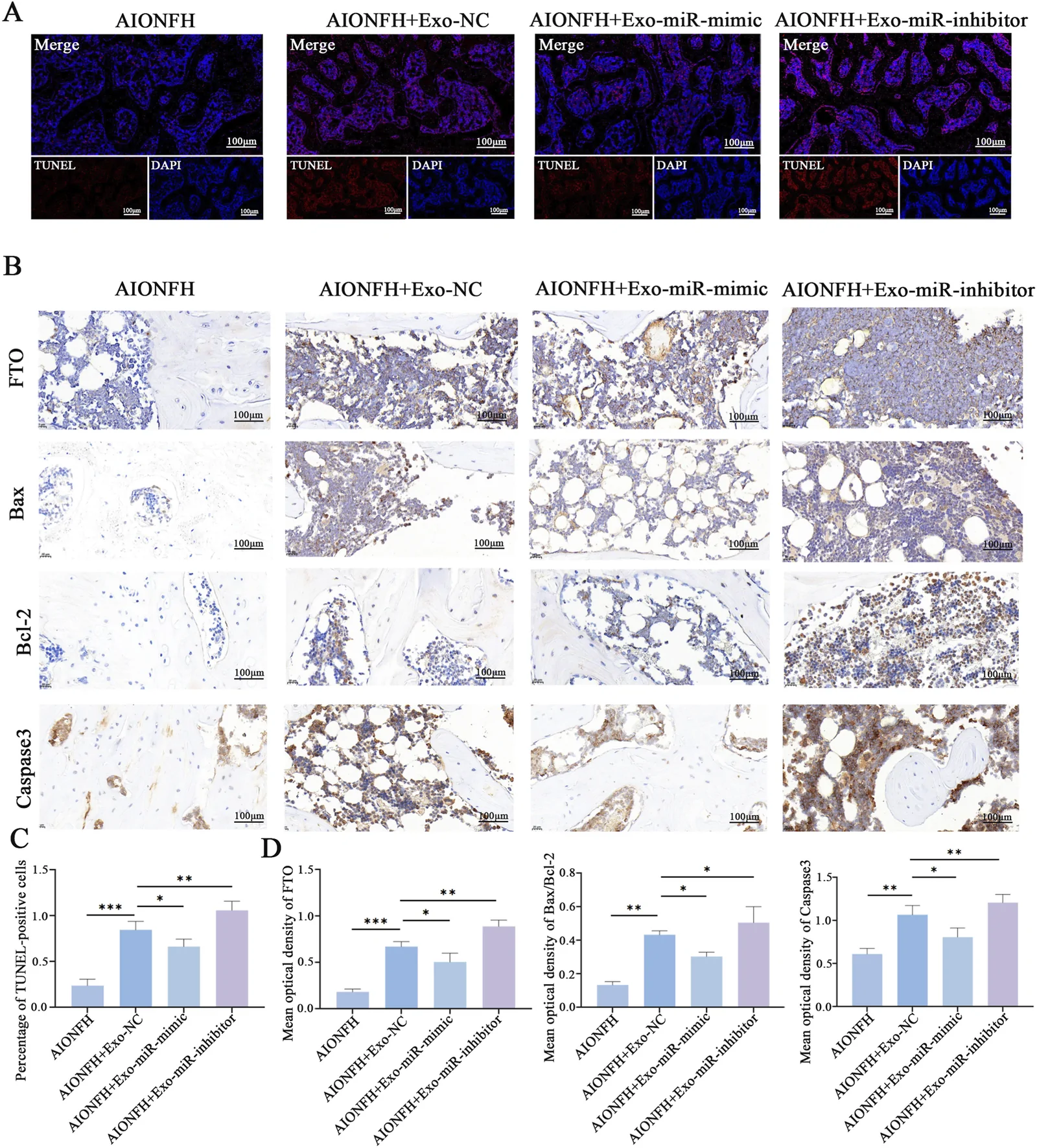
Osteoclast-derived exosomal miR-378a-3p targets FTO to prevent AIONFH by inhibiting apoptosis *in vivo*. **(A)** TUNEL immunofluorescence staining (red) with DAPI nuclear counterstaining (blue) and merged images of femoral head tissue across the four groups. Scale bar = 100 μm. **(B)** Immunohistochemical staining of FTO, Bax, Bcl-2, and Caspase-3 across the four groups. Scale bar = 100 μm. **(C)** Quantification of TUNEL-positive cell percentage. **(D)** Mean optical density quantification of FTO, Bax/Bcl-2 ratio, and Caspase-3. n = 3; data are mean ± SD; *P < 0.05, **P < 0.01, ***P < 0.001.

## 4 Discussion

Bone metabolic balance is maintained by an equilibrium between osteoclast-mediated bone resorption and the repair capacity of BMSCs. Chronic alcohol exposure disrupts this balance and is a major cause of AIONFH (Chen et al., 2017). In this study, we established an AIONFH rat model and focused on trabecular bone regeneration and osteocyte survival in the femoral head subchondral region, as these are the main pathological features of early-stage AIONFH. We evaluated bone structure by Micro-CT and assessed osteocyte apoptosis and new bone formation by histological staining. Our findings agree with previous research showing that alcohol impairs bone balance by overactivating osteoclasts and reducing BMSC osteogenic potential, while also causing osteocyte apoptosis, which together accelerate bone death (Tang et al., 2025; Hemati et al., 2026). BMSC-mediated osteogenic differentiation is closely linked to the repair of the osteonecrotic femoral head. Improved osteogenic differentiation promotes new bone formation in the necrotic region and helps repair necrotic bone tissue (Zhang S. et al., 2025). Osteocyte apoptosis is closely linked to the progression and collapse of the osteonecrotic femoral head. Continued excessive osteocyte apoptosis disrupts bone structure and leads to femoral head collapse (Li et al., 2025). Recently, the role of osteoclast-released exosomes in the bone microenvironment has received more attention. These exosomes act as important carriers for long-distance cell communication, using encapsulated miRNAs to control cell functions and regulate bone remodeling through epigenetic pathways (Wang et al., 2022; Liu et al., 2024). However, the specific effects of osteoclast-derived exosomal miRNAs on m^6^A modifications under alcohol conditions remain poorly understood.

In our studies, alcohol exposure strongly promoted BMM maturation into osteoclasts. Through high-throughput screening, we identified two candidate miRNAs with different expression in alcohol-exposed exosomes: miR-378a-3p (significantly decreased) and miR-376a-3p (significantly increased). Previous studies have shown that both are involved in bone metabolism regulation: increased miR-376a-3p reduces the expression of osteogenic markers including ALP, Runx2, and OSX, which inhibits bone formation; miR-378a-3p is expressed at low levels in BMSCs from osteoporotic rats, and its overexpression increases osteogenic markers and promotes bone formation (Zhang J. et al., 2025; Zhang et al., 2018; Yu X. et al., 2020). We focused on miR-378a-3p for two reasons: bioinformatic analysis showed a potential binding site for miR-378a-3p in the FTO 3′-UTR, but no such site for miR-376a-3p; also, its decreased expression matches the impaired bone formation seen in AIONFH. However, although previous studies have linked miR-378a-3p to bone metabolism, how this miRNA is transported outside cells and what its downstream targets are remain unclear, especially in alcohol-induced injury and cell death. Our study provides new findings by showing that osteoclast-derived miR-378a-3p has a dual protective effect: it promotes bone repair and inhibits osteocyte apoptosis by targeting FTO to regulate m^6^A levels. Under alcohol stress, miR-378a-3p-containing exosomes not only maintain BMSC viability and restore their regenerative capacity but also protect mature osteocytes from apoptosis, reduce empty bone spaces, and maintain bone structure. We also used bioinformatic prediction and dual-luciferase reporter assays to confirm that miR-378a-3p binds to the FTO 3′-UTR and reduces its expression, filling the knowledge gap about the role of exosomal miRNAs in AIONFH.

As a key m^6^A demethylase, FTO regulates stem cell differentiation by changing the RNA methylation pattern (Chen et al., 2022; Zheng et al., 2024). Evidence shows that FTO overexpression can reduce RUNX2 expression, decrease m^6^A levels, and block BMSC differentiation (Wang J. et al., 2021). Our research found high FTO expression in BMSCs exposed to alcohol, which was linked to reduced osteogenic capacity and increased apoptosis. Reducing FTO successfully restored BMSC function, while increasing FTO worsened the harmful effects of alcohol. Using a dual-fluorescence tracking assay, we showed the transfer of miR-378a-3p from osteoclasts to BMSCs, indicating that osteoclast-derived exosomes are important carriers in this regulatory network. Follow-up rescue experiments confirmed this pathway: alcohol exposure increases FTO and reduces bone formation, while adding exosomes enriched with miR-378a-3p reverses this by reducing FTO and increasing m^6^A levels.

*In vivo*, the results matched these cell-based findings. Systemic injection of alcohol-exposed exosomes worsened femoral head necrosis, increased collagen destruction, and reduced bone mass in rats. In contrast, injection of exosomes overexpressing miR-378a-3p reduced the necrotic area, promoted subchondral bone regeneration, and significantly reduced tissue damage. Immunofluorescence and immunohistochemistry results confirmed that this treatment successfully reduced FTO, restored osteogenic markers like ALP and OCN, and reduced apoptotic indicators including TUNEL-positive ratio, Caspase-3 expression, and Bax/Bcl-2 protein ratio. Together, these results show a disease process where alcohol reduces osteoclast-derived miR-378a-3p, which increases FTO in BMSCs, leading to reduced m^6^A modification and impaired bone remodeling.

However, this study has several limitations. First, regarding exosome methods, contamination by non-exosomal particles and protein impurities during ultracentrifugation cannot be excluded, which may affect result interpretation. Also, *in vivo* assessment was limited to tail vein injection without direct evaluation of exosome distribution and targeting to the femoral head, and the uptake pathways and intracellular release mechanism of exosomal miR-378a-3p remain unclear. Future studies should use *in vivo* imaging and specific uptake inhibitors combined with live-cell imaging to address these issues. Second, therapeutic effects were assessed only at a single 6-week time point, making it difficult to separate short-term improvement from long-term repair. Future studies using multiple time points are needed. Third, the miR-378a-3p–FTO pathway was tested only through a rescue experiment with FTO overexpression, so other downstream targets or FTO-independent mechanisms cannot be ruled out. Future studies using FTO loss-of-function models and genome-wide target identification are needed. Fourth, m^6^A detection was limited to total RNA levels without separating mRNA from non-coding RNA modifications, so changes in total m^6^A content may not fully represent modifications on functional target mRNAs, and future genome-wide m^6^A sequencing is needed. Finally, all experiments were done in cell and rat models, and the clinical relevance needs to be tested in human specimens from AIONFH patients.

## 5 Conclusion

In summary, chronic alcohol exposure reduces miR-378a-3p in osteoclast-derived exosomes, leading to increased FTO expression, reduced m^6^A modification, impaired osteogenesis, and increased apoptosis. Exosomes enriched with miR-378a-3p reverse these effects and improve femoral head bone damage in AIONFH rats. The osteoclast-derived exosomal miR-378a-3p/FTO/m^6^A pathway may be a potential therapeutic target for AIONFH.

## Data Availability

The original contributions presented in the study are included in the article/Supplementary Material, further inquiries can be directed to the corresponding authors.

## References

[B1] Chen Y. X. Tao S. C. Xu Z. L. Yin W. J. Zhang Y. L. Yin J. H. (2017). Novel Akt activator SC-79 is a potential treatment for alcohol-induced osteonecrosis of the femoral head. Oncotarget 8 (19), 31065–31078. 10.18632/oncotarget.16075 28415692 PMC5458189

[B2] Chen L. S. Zhang M. Chen P. Xiong X. f. Liu P. q. Wang H. b. (2022). The m6A demethylase FTO promotes the osteogenesis of mesenchymal stem cells by downregulating PPARG. Acta Pharmacol. Sin. 43 (5), 1311–1323. 10.1038/s41401-021-00756-8 PMC9061799 34462564

[B3] Cheng M. Lu H. Wu Y. Jia L. Xiang T. Deng L. (2025). Sex differences in alcohol inhibits bone formation and promotes bone resorption in young Male and female rats by altering intestinal flora, metabolites, and bone microenvironment. PLoS One 20 (5), e0323222. 10.1371/journal.pone.0323222 PMC12061194 40338892

[B4] Hemati H. Blanton M. B. Koura J. Khadka R. Grant K. A. Messaoudi I. (2026). Chronic alcohol consumption enhances the differentiation capacity of hematopoietic stem and progenitor cells into osteoclast precursors. Am. J. Pathol. 196 (1), 326–343. 10.1016/j.ajpath.2025.06.010 40683559 PMC12799408

[B5] Hong G. Han X. He W. Xu J. Sun P. Shen Y. (2019). Analysis of circulating microRNAs aberrantly expressed in alcohol-induced osteonecrosis of femoral head. Sci. Rep. 9 (1), 18926. 10.1038/s41598-019-55188-6 31831773 PMC6908598

[B6] Huang M. Guo J. Liu L. Jin H. Chen X. Zou J. (2023). m6A demethylase FTO and osteoporosis: potential therapeutic interventions. Front. Cell Dev. Biol. 11, 1275475. 10.3389/fcell.2023.1275475 38020896 PMC10667916

[B7] Jia Y. Jiang J. Zhao K. Zhang T. Sun P. Peng J. (2019). Disulfiram suppressed ethanol promoted RANKL-induced osteoclastogenesis *in vitro* and ethanol-induced osteoporosis *in vivo* via ALDH1A1-NFATc1 axis. Aging (Albany NY) 11 (19), 8103–8119. 10.18632/aging.102279 31596733 PMC6814600

[B8] Konarski W. Poboży T. Konarska K. Śliwczyński A. Kotela I. Hordowicz M. (2023). Osteonecrosis related to steroid and alcohol Use-An update on pathogenesis. Healthc. (Basel) 11 (13), 1846. 10.3390/healthcare11131846 PMC10340773 37444680

[B9] Le G. Wen R. Fang H. Huang Z. Wang Y. Luo H. (2025). Exosomal miR-122 derived from M2 macrophages induces osteogenic differentiation of bone marrow mesenchymal stem cells in the treatment of alcoholic osteonecrosis of the femoral head. J. Orthop. Surg. Res. 20 (1), 107. 10.1186/s13018-025-05515-7 39881350 PMC11776149

[B10] Li C. Ji H. Zhuang S. Xie X. Cui D. Zhang C. (2025). Update on the correlation between mitochondrial function and osteonecrosis of the Femoral head osteocytes. Redox Rep. 30 (1), 2491846. 10.1080/13510002.2025.2491846 40249372 PMC12010656

[B11] Liao Z. Jin Y. Chu Y. Wu H. Li X. Deng Z. (2022). Single-cell transcriptome analysis reveals aberrant stromal cells and heterogeneous endothelial cells in alcohol-induced osteonecrosis of the femoral head. Commun. Biol. 5 (1), 324. 10.1038/s42003-022-03271-6 35388143 PMC8987047

[B12] Liu J. Wang B. Chen H. Yu X. Cao X. Zhang H. (2024). Osteoclast-derived exosomes influence osteoblast differentiation in osteoporosis progression via the lncRNA AW011738/miR-24-2-5p/TREM1 axis. Biomed. Pharmacother. 178, 117231. 10.1016/j.biopha.2024.117231 39094542

[B13] Maurel D. B. Pallu S. Jaffré C. Fazzalari N. L. Boisseau N. Uzbekov R. (2012). Osteocyte apoptosis and lipid infiltration as mechanisms of alcohol-induced bone loss. Alcohol Alcohol 47 (4), 413–422. 10.1093/alcalc/ags057 22596044

[B14] McGeary S. E. Lin K. S. Shi C. Y. Pham T. M. Bisaria N. Kelley G. M. (2019). The biochemical basis of microRNA targeting efficacy. Science 366 (6472), eaav1741. 10.1126/science.aav1741 31806698 PMC7051167

[B15] Pang K. Li H. Chen H. Man Z. Wang D. Li W. (2025). The bibliometric and visualization analysis of alcohol-induced osteonecrosis of the femoral head: research trends and future directions (1998-2024). J. Orthop. Surg. Res. 20 (1), 724. 10.1186/s13018-025-06138-8 40753253 PMC12317480

[B16] Parikh R. R. Mirzaei A. Butler M. E. Restrepo D. J. Guarin Perez S. F. Brandt S. (2026). Diagnosis and treatment of nontraumatic osteonecrosis of the femoral head: a systematic review and meta-analyses for the ARCO clinical practice guideline development workgroup. Med. Sci. (Basel) 14 (1), 107. 10.3390/medsci14010107 41892822 PMC13027876

[B17] Shuai Q. Tian J. Yang W. Huang S. (2026). Exosomes in osteoporosis: regulatory mechanisms and clinical applications. Front. Physiol. 17, 1792752. 10.3389/fphys.2026.1792752 PMC13106144 42038001

[B18] Sun W. Zhao C. Li Y. Wang L. Nie G. Peng J. (2016). Osteoclast-derived microRNA-containing exosomes selectively inhibit osteoblast activity. Cell Discov. 2, 16015. 10.1038/celldisc.2016.15 27462462 PMC4886818

[B19] Tan B. Li W. Zeng P. Guo H. Huang Z. Fu F. (2021). Epidemiological study based on China osteonecrosis of the femoral head database. Orthop. Surg. 13 (1), 153–160. 10.1111/os.12857 33347709 PMC7862166

[B20] Tang J. Wang X. Lin X. Wu C. (2024). Mesenchymal stem cell-derived extracellular vesicles: a regulator and carrier for targeting bone-related diseases. Cell Death Discov. 10 (1), 212. 10.1038/s41420-024-01973-w 38697996 PMC11066013

[B21] Tang Z. Xu X. Shi W. Ren X. Luo H. Xu Y. (2025). Huc-MSC-derived exosomes alleviates alcohol-induced osteonecrosis of the femoral head through targeting the miR-25-3p/GREM1 axis. Genomics 117 (2), 110996. 10.1016/j.ygeno.2025.110996 39826815

[B22] Wang X. K. Zhang Y. W. Wang C. M. Li B. Zhang T. Z. Zhou W. J. (2021). METTL16 promotes cell proliferation by up-regulating cyclin D1 expression in gastric cancer. J. Cell Mol. Med. 25 (14), 6602–6617. 10.1111/jcmm.16664 PMC8278090 34075693

[B23] Wang J. Fu Q. Yang J. Liu J. L. Hou S. M. Huang X. (2021). RNA N6-methyladenosine demethylase FTO promotes osteoporosis through demethylating Runx2 mRNA and inhibiting osteogenic differentiation. Aging (Albany NY) 13 (17), 21134–21141. 10.18632/aging.203377 34496349 PMC8457567

[B24] Wang Z. Zhao Z. Gao B. Zhang L. (2022). Exosome mediated biological functions within skeletal microenvironment. Front. Bioeng. Biotechnol. 10, 953916. 10.3389/fbioe.2022.953916 35935491 PMC9355125

[B25] Wang C. Wang P. Li F. Li Y. Zhao M. Feng H. (2023). Study on the association of the microstructure and bone metabolism in the osteoporotic femoral head. Mol. Biol. Rep. 50 (9), 7437–7444. 10.1007/s11033-023-08505-2 PMC10460722 37479877

[B26] Wang Y. Wang H. Tan J. Cao Z. Wang Q. Wang H. (2025). Therapeutic effect of mesenchymal stem cells and their derived exosomes in diseases. Mol. Biomed. 6 (1), 34. 10.1186/s43556-025-00277-4 40465163 PMC12137892

[B27] Wu Z. Li W. Jiang K. Lin Z. Qian C. Wu M. (2020). Regulation of bone homeostasis: signaling pathways and therapeutic targets. MedComm 5 (8), e657. 10.1002/mco2.657 PMC11266958 39049966

[B28] Xie X. Li C. Zheng H. Gan G. Gao X. (2026). m6A modification in skeletal system diseases. Mol. Med. Rep. 34 (1), 198. 10.3892/mmr.2026.13908 PMC13220071 42169641

[B29] Ye Q. Y. Cui Y. Wang H. Y. Li L. Y. Chen J. B. Zhu X. F. (2024). Exosomal communication: a pivotal regulator of bone homeostasis and a potential therapeutic target. Front. Pharmacol. 15, 1516125. 10.3389/fphar.2024.1516125 39764467 PMC11700997

[B30] Yoon B. H. Jones L. C. Chen C. H. Cheng E. Y. Cui Q. Drescher W. (2019). Etiologic classification criteria of ARCO on femoral head osteonecrosis part 2: alcohol-associated osteonecrosis. J. Arthroplasty 34 (1), 169–174.e1. 10.1016/j.arth.2018.09.006 30348559

[B31] Yu H. Zhu D. Liu P. Yang Q. Gao J. Huang Y. (2020). Osthole stimulates bone formation, drives vascularization and retards adipogenesis to alleviate alcohol-induced osteonecrosis of the femoral head. J. Cell Mol. Med. 24 (8), 4439–4451. 10.1111/jcmm.15103 32135036 PMC7176840

[B32] Yu X. Zeng Y. Bao M. Wen J. Zhu G. Cao C. (2020). Low-magnitude vibration induces osteogenic differentiation of bone marrow mesenchymal stem cells via miR-378a-3p/Grb2 pathway to promote bone formation in a rat model of age-related bone loss. FASEB J. 34 (9), 11754–11771. 10.1096/fj.201902830RRR 32652777

[B33] Zhang B. Li Y. Yu Y. Zhao J. Ou Y. Chao Y. (2018). MicroRNA-378 promotes osteogenesis-angiogenesis coupling in BMMSCs for potential bone regeneration. Anal. Cell Pathol. (Amst) 2018, 8402390. 10.1155/2018/8402390 29686962 PMC5852880

[B34] Zhang S. Wang H. Meng Q. Lee W. Y. Li Z. Sun S. (2025). Recent advances in osteonecrosis of the femoral head: a focus on mesenchymal stem cells and adipocytes. J. Transl. Med. 23 (1), 592. 10.1186/s12967-025-06564-6 40426076 PMC12108002

[B35] Zhang J. Zhang J. Zhang F. Jing S. Yang D. (2025). MiR-376a-3p inhibits bone repair by regulating osteoblastic differentiation. J. Orthop. Surg. Res. 20 (1), 887. 10.1186/s13018-025-06291-0 41084006 PMC12516833

[B36] Zheng J. Lu Y. Lin Y. Si S. Guo B. Zhao X. (2024). Epitranscriptomic modifications in mesenchymal stem cell differentiation: advances, mechanistic insights, and beyond. Cell Death Differ. 31 (1), 9–27. 10.1038/s41418-023-01238-6 37985811 PMC10782030

