# Supplementary material for: Osteoclast-derived exosomal miR-378a-3p targets FTO-mediated m6A modification to promote osteogenesis and inhibit osteocyte apoptosis in alcohol-induced osteonecrosis of the femoral head
Source: Front Cell Dev Biol. 2026 Oct 26;14:1975355. doi: 10.3389/fcell.2026.1975355 (PMC13745732; doi:10.3389/fcell.2026.1975355)
Supplement: Supplementary file 1 [file Table1.docx]

## Supplementary Materials

**Figure S1: Fluorescence microscopic images after transfection with miR-378a-3p overexpression/knockdown plasmids and RT-qPCR detection of miR-378a-3p expression efficiency in each group.**

A: Fluorescence microscopic images (100×) after transfecting BMDMs with miR-mimic, miR-inhibitor, and their respective negative controls (miR-mimic-NC, miR-inhibitor-NC). B: After transfection, the expression efficiency of miR-378a-3p in BMDMs of each group was detected by RT-qPCR. **: *P* < 0.01; ***: *P* < 0.001.

**Figure S2: Fluorescence microscopic images after transfection with FTO-overexpressing plasmids/FTO-knockdown siRNAs and RT-qPCR detection of FTO expression efficiency in each group.** A: Fluorescence microscopic images (100×) after transfecting BMSCs with si-FTO, oe-FTO, and their respective negative controls (si-NC, Vector-NC). B: After transfection, the expression efficiency of FTO in BMSCs of each group was detected by RT-qPCR. ***: *P* < 0.001.

**Table ST1: Information on the dual-luciferase PmirGLO vectors**

| **Name** | **Construction Sequence** | **Vector** |
| --- | --- | --- |
| FTO-WT | AGGATGCACCAGTCCTAGGTGGAGAAGGAAAAGATGTCCTTGGCTTTTCTCCCCAGTCCTTG | PmirGLO |
| FTO-MUT | AGGATGCACCAGTCCGTCCATCAGAAGCTTAAGATGTCCGACCCTTTTCTCCCCAGTCCTTG | PmirGLO |

**Table ST2: miRNA primers (stem-loop method)**

| **Gene** | **Primer type** | **Primer name** | **Sequence (5′→3′)** | **Amplicon (bp)** |
| --- | --- | --- | --- | --- |
| miR-376a-3p | RT primer | miR-376a-3p-RT | GTCGTATCCAGTGCAGGGTCCGAGGTATTCGCACTGGATACGACACGTGG | — |
| miR-376a-3p | Forward | miR-376a-3p-F | GCGCGATCGTAGAGGAAAAT | 65 |
| miR-320-3p | RT primer | miR-320-3p-RT | GTCGTATCCAGTGCAGGGTCCGAGGTATTCGCACTGGATACGACTCGCCC | — |
| miR-320-3p | Forward | miR-320-3p-F | GCGAAAAGCTGGGTTGAGA | 66 |
| miR-378a-3p | RT primer | miR-378a-3p-RT | GTCGTATCCAGTGCAGGGTCCGAGGTATTCGCACTGGATACGACCCTTCT | — |
| miR-378a-3p | Forward | miR-378a-3p-F | CGCGACTGGACTTGGAGTC | 65 |
| Universal | Reverse | Universal-R | AGTGCAGGGTCCGAGGTATT | — |

**Table ST3:Reference genes**

| **Gene** | **Primer name** | **Sequence (5′→3′)** | **Amplicon (bp)** |
| --- | --- | --- | --- |
| U6 | U6-F | CTCGCTTCGGCAGCACAT | 102 |
| U6 | U6-R | AAATATGBAACGCTTCACG |  |
| β-actin | β-actin-F | GGCTGTATTCCCCTCCATCG | 154 |
| β-actin | β-actin-R | CCAGTTGGTAACAATGCCATGT |  |

**Table ST4:mRNA‑specific primers**

| **Gene** | **Primer name** | **Sequence (5′→3′)** | **Amplicon (bp)** |
| --- | --- | --- | --- |
| FTO | FTO-F | GTGTGACAAATGCCGTGCTT | 169 |
| FTO | FTO-R | TGCTGTGCTGGTAGAGTTCG |  |
| ALP | ALP-F | CGTGCATTCATTGTGCGACT | 141 |
| ALP | ALP-R | AAAGAGACCTCTGCCGCTC |  |
| OCN | OCN-F | CCGTTTAGGGCATGTGTTGC | 108 |
| OCN | OCN-R | CCGTCCATACTTTCGAGGCA |  |
| Collagen I | Collagen I-F | CATGGCCAAGAAGACATCCC | 142 |
| Collagen I | Collagen I-R | CTTTGCATAGCACGCCATCG |  |
| Bax | Bax-F | TTTGCTACAGGGTTTCATCCAG | 145 |
| Bax | Bax-R | GTTGTTGTCCAGTTCATCGCC |  |
| Bcl-2 | Bcl2-F | TTGTGGCCTTCTTTGAGTTCG | 151 |
| Bcl-2 | Bcl2-R | GCATCCCAGCCTCCGTTAT |  |
| Caspase-3 | Caspase-F | GAAAGCCGAAACTCTTCATCAT | 93 |
| Caspase-3 | Caspase-R | ATGCCATATCATCGTCAGTTCC |  |
| PUMA | PUMA-F | ACCAGCCCAGCAGCACCTAG | 173 |
| PUMA | PUMA-R | TTGTCTCCGCCGCTCGTAC |  |
